# Regulation of Laser-Deposited Silver Microstructures on Ceramic Surfaces and Their Effects on Electrical Conductivity

**DOI:** 10.3390/mi17060702

**Published:** 2026-06-08

**Authors:** Hui Zhang, Yongling Wu, Hongyu Zheng

**Affiliations:** Centre for Advanced Laser Manufacture, School of Mechanical Engineering, Shandong University of Technology, Zibo 255000, China; 17852034295@163.com (H.Z.);

**Keywords:** alumina ceramics, nanosecond laser-induced deposition, silver microstructures, interface regulation, conductive performance

## Abstract

Silver conductive structures were fabricated on 96% alumina ceramic substrates by selectively irradiating a silver nitrate precursor liquid film using a 355 nm Nd:YAG nanosecond laser under ambient conditions, without the use of external reducing agents. The effects of laser energy density, scan number, precursor concentration, plasma pretreatment, and PVP-30 addition on the morphology, composition, electrical conductivity, and adhesion of the deposited structures were investigated using XRD, SEM, EDS, contact angle measurements, resistance measurements, and tape-peeling tests. XRD confirmed the formation of metallic Ag in the laser-scanned regions. Insufficient laser energy density led to incomplete Ag^+^ reduction and discontinuous conductive paths, whereas excessive energy input caused hollow formation and Ag edge accumulation. A laser energy density of 12.03 J/cm^2^ provided a favorable balance among structural integrity, Ag enrichment, and electrical conductivity. Increasing the scan number promoted particle coalescence and conductive network formation, while 1000 scanning cycles provided a suitable balance between structural continuity and dimensional precision. As the AgNO_3_ concentration increased, the deposited structures evolved from isolated particles into continuous and compact layers, with 5 mol/L showing favorable deposition performance. Plasma pretreatment combined with PVP-30 addition reduced the contact angle of the ceramic surface from 48.25° to 19.05°, thereby improving the continuity, uniformity, and compactness of the deposits. After the scan spacing was reduced to form continuous silver films, the samples retained more than 98% of their conductivity after five tape-peeling cycles, with a resistivity of 6.14 × 10^−8^ Ω·m. These results demonstrate that laser-induced deposition is a controllable strategy for fabricating conductive silver structures on ceramic surfaces.

## 1. Introduction

Alumina ceramics possess excellent electrical insulation, high-temperature resistance, corrosion resistance, thermal stability, and mechanical strength, and are therefore widely employed in electronic packaging, power device substrates, microsensors, and high-temperature electronic devices [1]. In practical applications, alumina ceramics are often integrated with metallic functional structures in a wide range of devices, including ceramic–metal electrical insulators, pressure sensors, biomedical implants, X-ray tubes, batteries, burners, and other harsh-environment components [2]. These applications require reliable conductive or functional metallic structures on ceramic surfaces to enable electrical interconnection, sensing, heating, or signal transmission while maintaining the thermal and chemical stability of the ceramic substrate. Nevertheless, the intrinsically low electrical conductivity and strong chemical inertness of ceramic materials, together with their inadequate wettability and weak interfacial bonding with metallic layers [3], limit their direct application in conductive interconnect structures for highly integrated devices. Consequently, the controllable fabrication of high-quality metallic conductive structures on ceramic surfaces has become a major research focus in the functionalization of ceramic materials.

Currently, ceramic surface metallization techniques mainly include screen printing [4,5,6], magnetron sputtering [7,8,9], chemical plating [10,11], and direct bonding copper (DBC) technology [12,13,14]. Although conventional methods can achieve metal coating on ceramic surfaces, they commonly suffer from drawbacks such as lengthy fabrication processes, strong dependence on masks, limited selectivity, difficulty in controlling interfacial bonding, and complicated post-treatment procedures. In contrast, laser-induced deposition technology offers unique advantages, including non-contact processing, localized fabrication, flexible patterning capability, high selectivity, and in situ manufacturing under ambient conditions. For instance, Li et al. [15] fabricated silver conductors on Al_2_O_3_ substrates via laser direct writing, achieving a minimum linewidth of approximately 20 μm. Hui et al. [16] developed a novel approach for copper metallization on alumina ceramic surfaces, in which the metallized layer mainly consisted of copper spheres with diameters of approximately 60 μm embedded within the substrate, together with a thin CuAlO_2_ interfacial layer, resulting in strong interfacial bonding and an almost indistinguishable interface. In addition to alumina ceramics, laser-assisted processes can also be employed to fabricate metallic structures on other ceramic substrates. Cheng et al. [17] developed a hybrid laser process for preparing thick silver coatings on AlN substrates, in which an aluminum layer was first induced on the AlN surface and subsequently laser-sintered with a silver layer, ultimately achieving thick silver functional coatings with a line resistance as low as 0.26 Ω. Nedyalkov et al. [18] further applied laser-induced conductive lines on AlN ceramic surfaces to integrate resistive heating elements and systematically investigated the effects of laser parameters on the electrical conductivity and thermal response characteristics. These studies demonstrate that laser direct writing technology not only provides high patterning precision for the selective fabrication of microscale metallic structures on ceramic surfaces but also exhibits excellent substrate adaptability and process scalability, enabling the fabrication of various metallic structures, including silver and copper, on diverse ceramic substrates. Furthermore, optimization of the processing parameters can further enhance the electrical conductivity, interfacial bonding strength, and functional performance of the metallic structures, thereby extending their applications from simple conductive pathways to thick-film functional layers and microdevices.

Accordingly, the fabrication of metallic structures on 96% alumina ceramic substrates was investigated through selective scanning of a silver nitrate precursor liquid film using a 355 nm nanosecond laser, enabling the in situ preparation of silver conductive structures under ambient conditions. The effects of laser energy density, scan number, and silver nitrate concentration on the morphological evolution, elemental distribution, and electrical properties of the deposited structures were systematically investigated. In addition, plasma pretreatment and the incorporation of PVP-30 were employed to optimize the ceramic/silver interfacial condition and the deposition solution system, with the aim of enhancing the continuity, compactness, and adhesion strength of the deposited layer. Furthermore, the interfacial adhesion stability and electrical performance of the samples were evaluated through tape-peeling and resistivity measurements, thereby establishing the relationship between processing parameters, structural morphology, and performance response.

## 2. Experimental Procedures

### 2.1. Materials and Experimental Equipment

**Figure 1:**
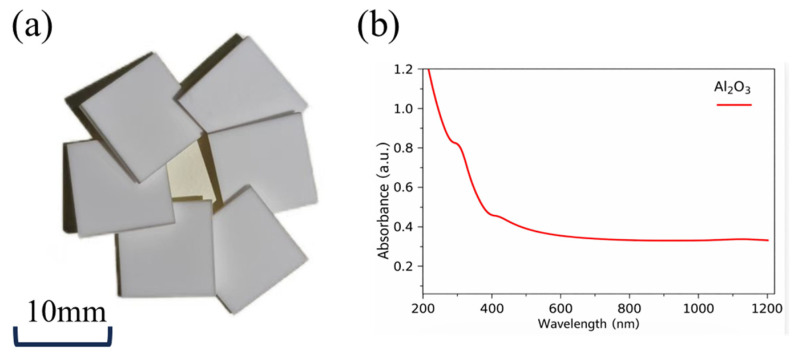
(**a**) photograph of the alumina ceramic specimen; (**b**) optical absorption spectrum of the alumina ceramic substrate measured in this work at different wavelengths, including the laser wavelength used in this study.

In this study, 96% alumina ceramic plates with dimensions of 10 mm × 10 mm × 1 mm were employed as the substrates for the deposition of silver conductive structures, as illustrated in Figure 1a. The chemical composition of the 96% alumina ceramic substrate is listed in Table 1. The substrate consisted primarily of Al_2_O_3_ (95.8 wt.%), with minor amounts of SiO_2_ (1.8 wt.%), MgO (1.4 wt.%), P_2_O_3_ (0.8 wt.%), and CaO (0.2 wt.%).

Considering the wavelength-dependent laser absorption behavior of the material, optical absorption measurements were performed on the alumina ceramic substrates at different wavelengths. Figure 1b presents the optical absorption spectrum measured in this work. As shown in Figure 1b, the substrate exhibited measurable optical absorption at 355 nm, corresponding to the wavelength of the laser system used in this study.

A Coherent Nd:YAG AVIA-LX nanosecond laser system (Coherent Inc., Santa Clara, CA, USA) was employed in the experiments, and the main laser parameters are summarized in Table 2. In addition, silver nitrate crystals were dissolved to prepare precursor solutions with different concentrations as the metal source [19], thereby supplying the silver ions required for metallic particle deposition. Polyvinylpyrrolidone (PVP) was introduced into the deposition solution as both a binder and dispersant to promote the uniform dispersion of silver ions within the precursor solution, suppress particle agglomeration, and improve the wettability and spreading behavior of the solution on the ceramic surface [20,21].

### 2.2. Experimental Procedure

Prior to the experiments, precursor solutions with different concentrations were prepared by dissolving various amounts of silver nitrate crystals in glass containers and adding deionized water to a final volume of 2 mL. After thorough mixing, silver nitrate precursor solutions with concentrations ranging from 0.5 to 7 mol/L were obtained. Subsequently, the 5 mol/L silver nitrate solution was divided into two portions. One portion was used directly as the PVP-30-free Ag-containing precursor for the experiments discussed in Section 3.1.1, Section 3.1.2 and Section 3.1.3, including the investigations of laser fluence, scan number, and Ag^+^ concentration. The other portion was modified by adding 0.2 wt.% PVP-30 and was used only for the interface engineering experiments discussed in Section 3.2. The ceramic substrates were subsequently ultrasonically cleaned in absolute ethanol for 15 min, rinsed, and further ultrasonically cleaned in deionized water for 10 min. The samples were then dried at 70 °C for 10 min to ensure clean and dry surfaces free from residual moisture. After the cleaning process, the ceramic substrates were placed on the plasma treatment stage. Physical bombardment and chemical reactions induced by high-energy particles within the plasma system enhanced the surface roughness and hydrophilicity of the ceramic substrates, thereby increasing the contact area between the substrates and the deposition solution. Finally, 0.1 mL of the pre-prepared deposition solutions with different compositions was dispensed onto the modified ceramic surface using a micro-droplet dispensing device, thereby forming a uniform and stable liquid film.

The laser deposition patterns were designed using EZCAD 2.7 software, and the corresponding laser processing parameters and scanning paths were configured accordingly. In this study, a rectangular raster-scanning pattern was adopted, in which the laser beam moved along parallel bidirectional scan lines in a serpentine manner with a fixed scan spacing of 0.1 mm. The scanning speed refers to the velocity of the laser spot along each scan line, whereas the scan spacing refers to the distance between two adjacent parallel scan lines. One scan, also referred to as one scanning cycle, was defined as one complete raster pass over the predefined rectangular processing area. Therefore, the scan number represents the number of repeated raster passes performed over the same area under identical scanning speed and scan spacing conditions.

**Figure 2:**
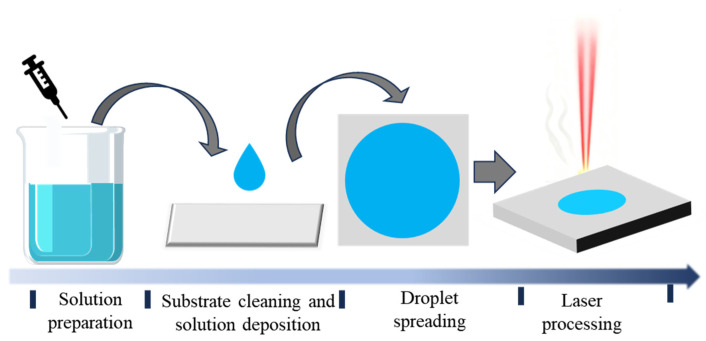
Schematic of the Experimental Process for Silver Conductive Structure Deposition on Ceramic Surfaces.

Under ambient atmospheric conditions at 25 °C, a nanosecond pulsed laser with a wavelength of 355 nm, a repetition rate of 30 kHz, and a scanning speed of 200 mm/s was employed to irradiate the pre-deposited liquid films under various preset processing conditions. The laser energy density ranged from 1.61 to 16.04 J/cm^2^, while the number of scanning cycles varied from 500 to 2000. After laser processing, the sample surfaces were first rinsed with deionized water to remove residual precursor solution, subsequently immersed in absolute ethanol for 10 min, and finally dried in a vacuum drying oven. For the quantitative linewidth and electrical resistance measurements, five repeated measurements were performed for each processing condition. The linewidth of each deposited structure was measured at five different positions along the laser-scanned region, and the electrical resistance was measured five times using a digital multimeter. The plotted data are presented as mean values, and the error bars represent the corresponding standard deviations. A schematic illustration of the overall experimental procedure is presented in Figure 2.

### 2.3. Instruments and Characterization

A Quanta 250 FEG field-emission scanning electron microscope (SEM, FEI Company, Hillsboro, OR, USA) was employed to characterize the microstructural morphology of the samples, with particular emphasis on the deposited microstructures on the ceramic surface. Furthermore, qualitative and quantitative compositional analyses of the deposited surfaces were performed using energy-dispersive spectroscopy (EDS, FEI Company, Hillsboro, OR, USA) integrated with the SEM system. Optical microscopy (OM, BX53MTRF-S, Olympus Corporation, Tokyo, Japan)was employed to examine the surface morphology of the samples, enabling direct observation of the macroscopic distribution, line continuity, and surface smoothness of the conductive silver structures, while also facilitating analysis of the effects of laser processing parameters on the structural morphology. A confocal laser scanning microscope (VK-250K, Keyence Corporation, Osaka, Japan) was utilized to obtain three-dimensional morphological information of the deposited structures. X-ray diffraction (XRD) analysis was performed using a D8 Advance X-ray powder diffractometer (Bruker AXS GmbH, Karlsruhe, Germany) to evaluate the diffraction peak positions, peak profiles, and relative intensities, thereby identifying the crystalline characteristics of the alumina substrate, verifying the formation of crystalline Ag phases after deposition, and determining whether different treatment conditions altered the primary crystal structure of the samples. The wettability of the sample surfaces was characterized using a JC2000C contact angle measurement system to evaluate the effects of ceramic surface wettability on the deposited structures. For electrical characterization, the resistance of the deposited structures was measured using a digital multimeter. Adhesion performance was evaluated using a Scotch™ 3M tape-peeling method in accordance with the ASTM D3359 standard [22] to investigate the interfacial bonding behavior and failure characteristics between the deposited structures and the alumina ceramic substrate.

## 3. Results and Discussion

### 3.1. Formation Mechanism of Laser-Deposited Silver Conductive Structures

**Figure 3:**
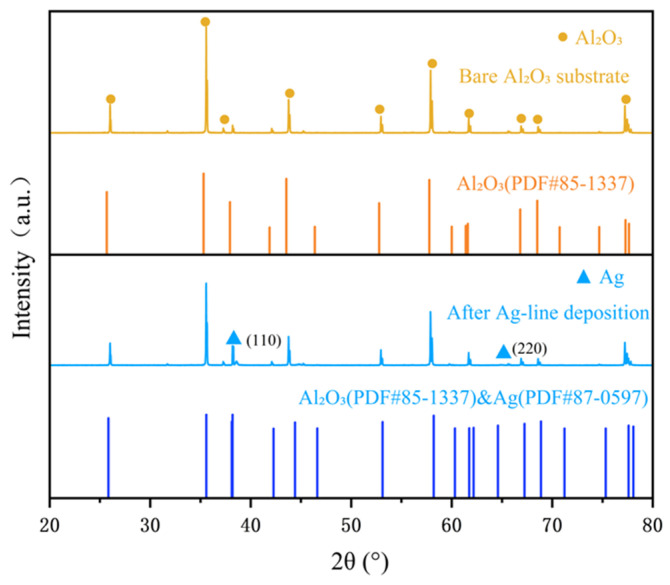
Comparison of the XRD patterns of alumina ceramic surface samples before and after laser deposition.

XRD analysis was performed on both the alumina ceramic substrate and the deposited sample surfaces, and the corresponding results are presented in Figure 3. In this section, the deposited sample was prepared using a 3 mol/L AgNO_3_ aqueous solution as the precursor, corresponding to an Ag^+^ concentration of 3 mol/L. New diffraction peaks appeared at 38.1° and 64.4°, which can be indexed to the (111) and (220) crystal planes of metallic Ag according to the standard PDF cards of α-Al_2_O_3_ (PDF#85-1337) and Ag (PDF#87-0597). The diffraction peaks corresponding to Ag were significantly weaker than those of alumina, suggesting that the deposited silver structures possessed relatively small thicknesses and a discontinuous distribution.

**Figure 4:**
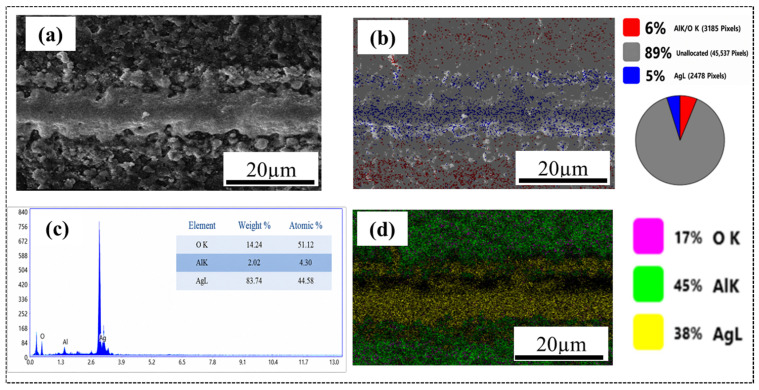
SEM images of the silver micro-lines and EDS elemental spectra of the deposited structures.

As revealed by the SEM and EDS results shown in Figure 4, laser scanning generated a relatively continuous strip-shaped deposited structure along the scanning path on the alumina ceramic surface. The central region of the deposited structure exhibited a relatively smooth morphology and good continuity, with a width of approximately 15 μm, and the deposited layer was primarily composed of silver. In addition, a considerable number of nanoparticles were distributed on both sides of the scanning path, with particle sizes ranging unevenly from 40 nm to 8 μm.

**Figure 5:**
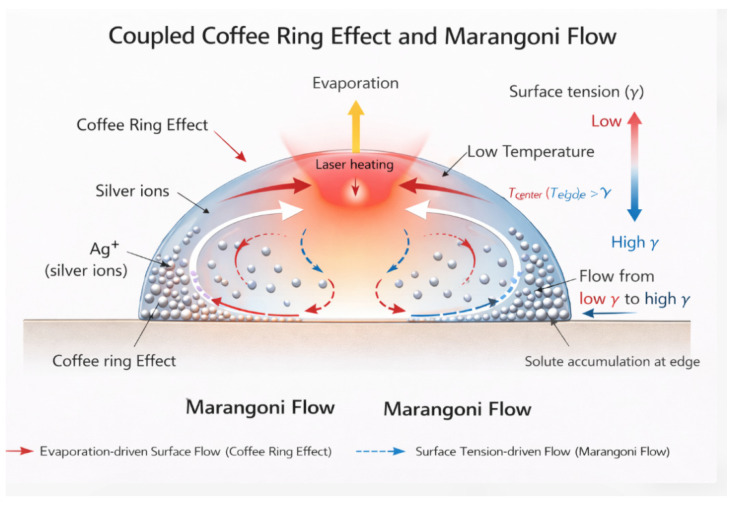
Schematic diagram of Ag^+^ migration in the droplet during laser-induced deposition, drawn based on the mechanism of droplet evaporation-induced solute redistribution reported in Ref. [25].

This behavior arises from the fact that the fabrication of metallic structures on ceramic surfaces through laser-induced reduction and deposition of metal ions fundamentally involves a complex multi-field coupling process [23]. On the one hand, the laser energy emitted by the experimental laser system exhibits an approximately Gaussian distribution [24], resulting in distinct laser-induced photothermal effects in different regions. Meanwhile, the localized laser-induced thermal field governs the transient temperature rise, evaporation behavior, and fluid flow within the precursor liquid film. Furthermore, evaporation-induced mass transport, temperature gradients, and surface tension gradients within the liquid film give rise to coffee-ring effects and Marangoni convection [25]. These interfacial flow behaviors can promote the migration of Ag^+^ ions and newly reduced Ag nanoparticles toward the periphery of the irradiated region or the solid–liquid–air contact line, resulting in nonuniform particle redistribution and edge accumulation. In addition, the migration and accumulation of Ag-containing species are not solely governed by bulk liquid flow, but may also involve complex interfacial phenomena, including nanoparticle migration, condensation, and assembly near the solid–liquid–air triple-phase region. Liu et al. reported that hybrid nanowires can self-assemble at fluid interfaces to form stable interfacial structures, demonstrating the important role of interfacial transport and assembly in regulating nanoscale material distribution [26]. Therefore, the Ag-rich edge accumulation and nonuniform deposited morphology observed in this work can be reasonably attributed to the coupled effects of laser-induced thermal gradients, evaporation-driven mass transport, Marangoni convection, and interfacial migration/condensation of Ag-containing species. Based on this mechanism, the migration behavior of Ag^+^ ions during the deposition process is schematically illustrated in Figure 5. These results further demonstrate that both the laser energy density and the spatial distribution of silver ions play critical roles in the reduction and deposition processes of silver micro-lines.

#### 3.1.1. Influence of Laser Energy Density on Deposition Products

**Figure 6:**
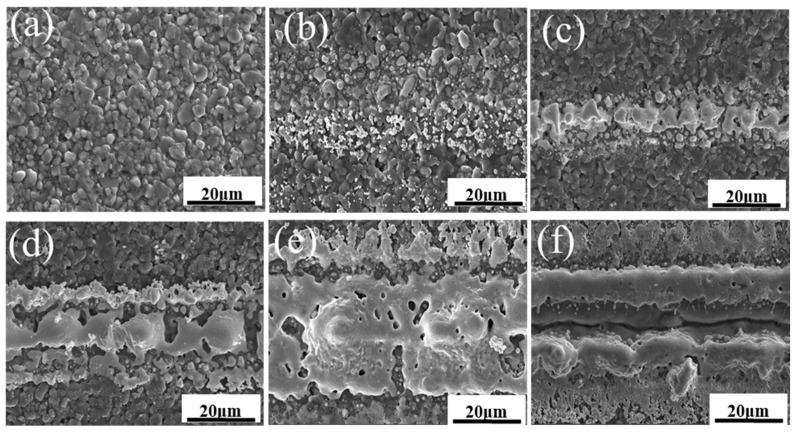
Effect of laser fluence on the morphology of the deposited products: SEM images of (**a**) ceramic substrate; (**b**) 1.61 J/cm^2^; (**c**) 4.01 J/cm^2^; (**d**) 8.02 J/cm^2^; (**e**) 12.03 J/cm^2^; and (**f**) 16.04 J/cm^2^.

**Figure 7:**
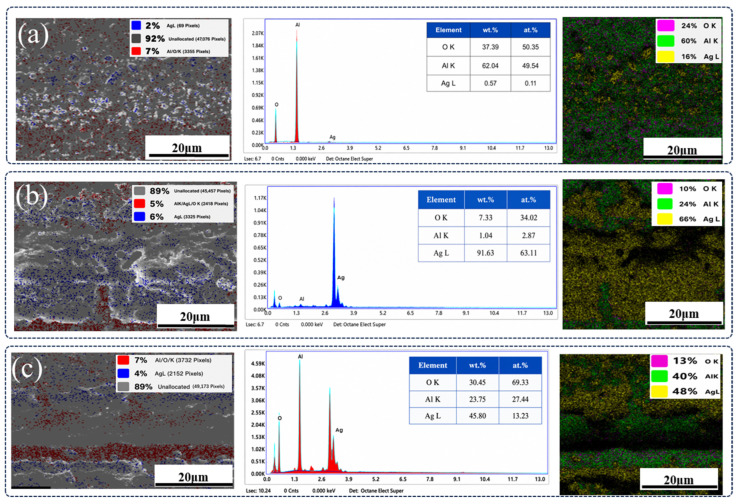
EDS spectra and elemental mapping results of the deposited structures under representative laser fluences: (**a**) low fluence, 1.61 J/cm^2^; (**b**) medium fluence, 12.03 J/cm^2^; and (**c**) high fluence, 16.04 J/cm^2^. For each condition, the left image shows the selected SEM/EDS analysis region, the middle graph shows the corresponding EDS spectrum and quantitative elemental composition, and the right image shows the elemental mapping results of Ag, Al, and O.

**Figure 8:**
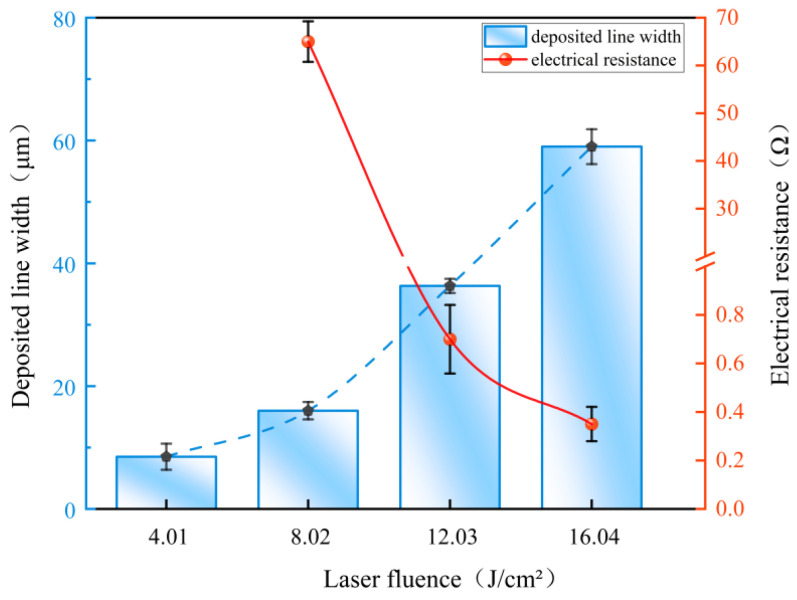
Effect of laser fluence on the width and resistance of the deposited structures.

To further investigate the influence of laser energy density on the morphology, composition, and electrical properties of the deposited products, experiments were conducted using a single-factor experimental design in which laser energy density was treated as the only variable. In this section, the Ag-containing precursor concentration was kept constant using a 5 mol/L AgNO_3_ aqueous solution, corresponding to an Ag^+^ concentration of 5 mol/L. Figure 6 presents the SEM morphological evolution of the deposited products fabricated under different laser energy densities. Figure 7 presents the EDS spectra and elemental mapping results of the deposited structures under representative laser fluences. The low-, medium-, and high-fluence conditions correspond to 1.61, 12.03, and 16.04 J/cm^2^, respectively. For each condition, the left image shows the selected SEM/EDS analysis region, the middle panel presents the EDS spectrum and quantitative elemental composition, and the right image shows the elemental mapping results for Ag, Al, and O. The EDS spectra provide quantitative elemental compositions of the selected regions in terms of weight and atomic percentages, whereas the elemental mapping images mainly reflect the spatial distribution or pixel-based classification of elemental signals. The corresponding variations in linewidth and electrical resistance are shown in Figure 8.

At a low laser energy density of 1.61 J/cm^2^, only a limited number of fine particles were generated, as shown in Figure 6b. According to the EDS analysis shown in Figure 7a, the surface was primarily composed of Al and O elements, whereas the Ag content remained extremely low at approximately 0.57 wt%, indicating insufficient reduction of silver ions and suggesting that the deposited products were predominantly in the initial nucleation stage. When the laser energy density was increased to 4.01 J/cm^2^, the laser scanning tracks became clearly distinguishable, as shown in Figure 6c. More particles accumulated locally along the laser path, forming deposited lines with widths of approximately 7 μm. Nevertheless, the deposited structure remained relatively porous and discontinuous, and a continuous conductive network was not established. Upon increasing the laser energy density to 8.02 J/cm^2^, the deposited bands became significantly more distinct, as shown in Figure 6d. Enhanced interparticle connectivity and noticeable local fusion were observed, with the linewidth increasing to approximately 15 μm. In addition, a local resistance of approximately 68 Ω was detected, indicating that the system had entered a critical transition stage from isolated particles to continuous conductive structures.

At a laser energy density of 12.03 J/cm^2^, a continuous deposited band with a width of approximately 37 μm was formed in the scanning region, as shown in Figure 6e, and the compactness of the deposited structure was markedly enhanced. In addition, Figure 7b reveals significant enrichment of Ag within the scanning region, accompanied by a substantial decrease in the contents of O and Al, indicating that the silver ions in the precursor solution were nearly completely reduced and that the deposited layer was primarily composed of metallic Ag. The resistance measured under this condition decreased to approximately 0.5 Ω, indicating that the laser energy input was well matched to the deposition process, thereby facilitating the formation of continuous, integrated, and compact silver conductive structures. When the laser energy density was further increased to 16.04 J/cm^2^, although the linewidth increased to 61 μm and the resistance decreased to 0.3 Ω, pronounced groove-like and hollow structures appeared in the center of the scanning region, as shown in Figure 6f. Meanwhile, silver particles preferentially accumulated on both sides of the laser scanning path, leading to pronounced edge accumulation. Consequently, the conductive network was mainly distributed along both sides of the scanning track, whereas the central region exhibited void areas lacking conductive structures. The corresponding EDS results shown in Figure 7c demonstrate that the Ag content in the scanning region decreased, whereas the signals of substrate elements such as O and Al became enhanced again, with Ag predominantly enriched along both sides of the scanning path. These findings suggest that excessively high laser energy density is not necessarily favorable for the formation of silver structures, as excessive energy input may damage the previously formed continuous deposited layer, leading to deposition deviation and structural instability.

In summary, insufficient laser energy density leads to inadequate reduction of silver ions, thereby hindering the formation of continuous conductive structures. In contrast, excessively high energy density causes overheating, central hollow formation, and instability of the deposited layer. Although the resistance further decreased at 16.04 J/cm^2^ owing to pronounced Ag accumulation along both sides of the scanning path, the central region exhibited void areas lacking conductive structures. Therefore, the laser energy density of 12.03 J/cm^2^ should not be regarded as the condition with the lowest resistance; rather, it represents a condition that provides a better balance among morphological integrity, dimensional uniformity, Ag enrichment, and electrical conductivity.

#### 3.1.2. Influence of Laser Scan Number on Deposition Products

**Figure 9:**
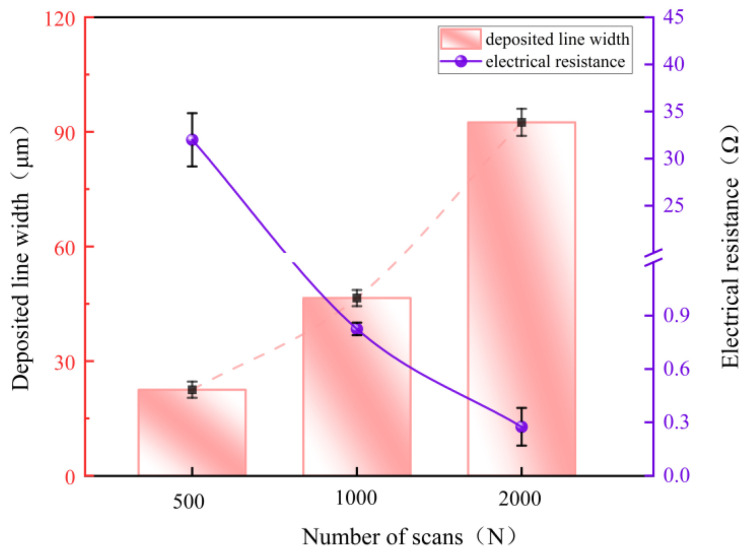
Effect of the number of scans on the line width and resistance of the deposited structures.

**Figure 10:**
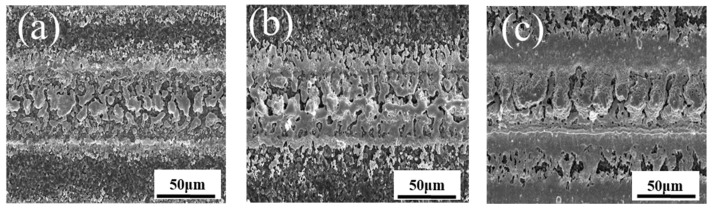
Effect of the number of laser scans on the morphology of the deposited products: SEM images of (**a**) 500 scans; (**b**) 1000 scans; and (**c**) 2000 scans.

Furthermore, in addition to controlling the laser energy intensity, the cumulative energy effect and laser–material interaction time can also be regulated by varying the laser scan number. The Ag-containing precursor concentration was kept constant using a 5 mol/L AgNO_3_ aqueous solution, corresponding to an Ag^+^ concentration of 5 mol/L. As a result, the morphology, continuity, and electrical performance of the deposited structures can be effectively tailored. Figure 9 illustrates the effects of laser scan number on the linewidth and electrical resistance of the deposited structures. Figure 10 presents SEM images showing the morphological evolution of the deposited products under different laser scan numbers.

Under a relatively short laser interaction duration corresponding to 500 scanning cycles, the deposited products within the scanning region appeared highly dispersed, with weak interparticle connectivity, noticeable pores, and poor surface uniformity, as illustrated in Figure 10a. As shown in Figure 9, the linewidth under this condition was approximately 34 μm, while the resistance remained as high as 23 Ω, suggesting that the deposited amount was insufficient and that a continuous conductive network had not yet been fully established. As the scan number increased to 1000, the deposited structures exhibited significantly improved continuity, stronger interparticle connectivity, and a smoother and more uniform surface morphology, as shown in Figure 10b. The linewidth increased to 48 μm, while the resistance decreased dramatically to 0.5 Ω, demonstrating that silver-ion reduction and deposition became more complete, thereby enabling the rapid formation of a continuous and stable conductive network. Upon further increasing the scan number to 2000, excessive lateral growth of the deposited structures occurred owing to thermal accumulation and diffusion effects, resulting in defects such as blurred and rough edges, a marked reduction in structural precision, and an increased linewidth of up to 98 μm, as shown in Figure 10c. Although the resistance further decreased to 0.3 Ω, the rate of reduction became considerably less pronounced.

In general, a moderate increase in laser interaction time contributes to enhanced continuity and improved electrical performance of the deposited structures. Nevertheless, excessively high scan numbers may lead to thermal accumulation and diffusion effects, resulting in excessive lateral growth, blurred and rough edges, and a significant decrease in structural precision. Although the resistance further decreased after 2000 scanning cycles, this improvement was accompanied by pronounced structural coarsening and reduced dimensional controllability. Therefore, under the investigated conditions, approximately 1000 scanning cycles provided a relatively better balance among structural continuity, dimensional precision, and electrical conductivity.

#### 3.1.3. Influence of Silver Ion Concentration on Deposition Products

**Figure 11:**
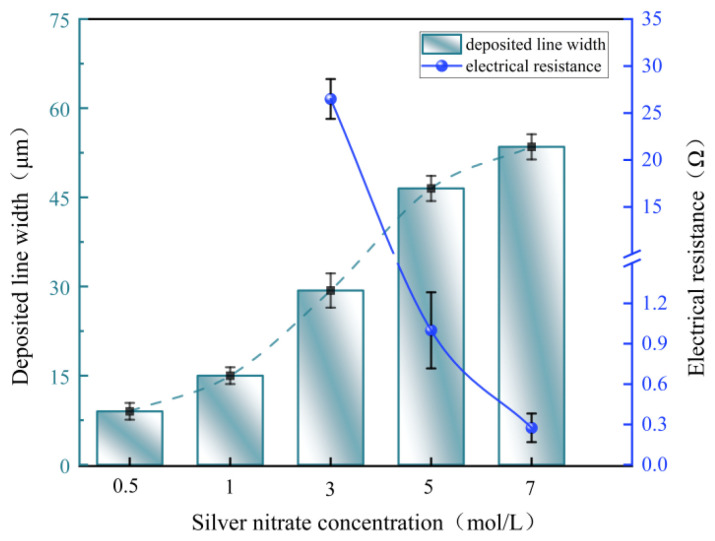
Effect of silver ion concentration on the width and resistance of the deposited structures.

**Figure 12:**
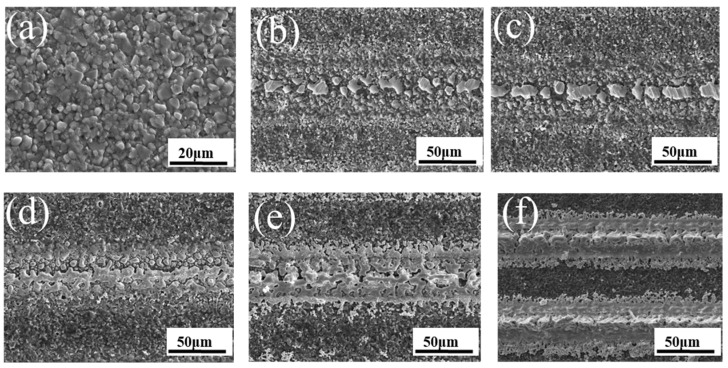
Effect of silver ion concentration on the morphology of the deposited products: SEM images of (**a**) ceramic substrate; (**b**) 0.5 mol/L; (**c**) 1 mol/L; (**d**) 3 mol/L; (**e**) 5 mol/L; and (**f**) 7 mol/L.

Following the investigation of laser energy intensity and laser interaction time, the effects of silver-ion concentration on the morphology and electrical performance of the deposited structures were further investigated. Figure 11 illustrates the variations in linewidth and electrical resistance of the deposited structures as a function of silver-ion concentration. Figure 12 presents the morphological evolution of the deposited structures under different silver-ion concentrations.

Figure 12a shows the surface morphology of the ceramic substrate. Under insufficient silver-ion supply conditions, namely at concentrations of 0.5 mol/L and 1 mol/L, the deposited products within the laser scanning region mainly consisted of dispersed fine particles exhibiting island-like or worm-like morphologies, as illustrated in Figure 12b,c. The particles were not effectively interconnected, and numerous pores and void regions remained, thereby preventing the formation of continuous line-like structures. In addition, reliable resistance measurements could not be obtained for samples prepared within this concentration range. As the concentration increased to 3 mol/L, the deposited bands became more clearly defined, as shown in Figure 12d. Interparticle contact gradually developed, and the microstructure evolved from isolated particle accumulation toward a continuous deposited layer with a linewidth of approximately 25 μm. The measured resistance under this condition was approximately 25 Ω, indicating that the Ag^+^ supply was sufficient to support the laser-induced deposition process, thereby improving interparticle connectivity and facilitating the gradual evolution of the deposited structure from a discontinuous state into a continuous conductive network.

When the concentration was further increased to 5 mol/L, the deposited structures exhibited optimal continuity and compactness, as shown in Figure 12e. The particles became tightly interconnected, the number of pores was significantly reduced, and the deposited layer appeared relatively uniform with well-defined boundaries. The linewidth rapidly increased from 25 μm to 48 μm, whereas the resistance sharply decreased from 25 Ω to 0.8 Ω. These results indicate that the silver-ion supply rate was well matched to the laser deposition rate under this condition, leading to significant improvements in deposition coverage, layer thickness, and interparticle bonding. Consequently, the conductive network rapidly evolved into a continuous and compact structure, and the electrical performance of the deposited structures was substantially enhanced. At a concentration of 7 mol/L, the deposition amount further increased; however, defects such as particle agglomeration, local protrusions, and edge roughening became evident, as shown in Figure 12f. The linewidth of the deposited structures further increased from 48 μm to 56 μm, while the resistance decreased only slightly from 0.8 Ω to 0.2 Ω. These findings suggest that, at excessively high concentrations, the improvement in electrical conductivity no longer scales proportionally with the increase in geometric dimensions, and the contribution of further increasing the silver-ion concentration to resistance reduction becomes progressively limited.

### 3.2. Interface Engineering and Performance Enhancement

Although the in situ fabrication of silver conductive structures on ceramic surfaces can be achieved by optimizing the laser energy intensity, interaction time, and silver-ion supply concentration, the deposited layers still suffer from insufficient interfacial bonding, loose structural morphology, and numerous local pores owing to the relatively smooth ceramic surface and the limited mechanical interlocking effect between the ceramic substrate and the metallic layer. In this section, a 5 mol/L AgNO_3_ aqueous solution was used as the Ag-containing precursor, corresponding to an Ag^+^ concentration of 5 mol/L. For comparison, both the PVP-30-free precursor solution and the precursor solution containing 0.2 wt.% PVP-30 was used to evaluate the effect of precursor solution modification. To address these issues, further optimization was conducted by regulating both the substrate surface condition and the precursor solution system.

**Figure 13:**
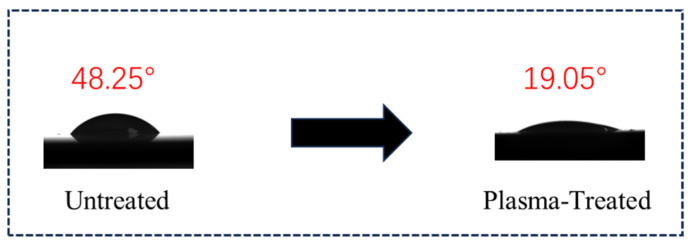
Contact angle variation in the ceramic surface after plasma pretreatment.

**Figure 14:**
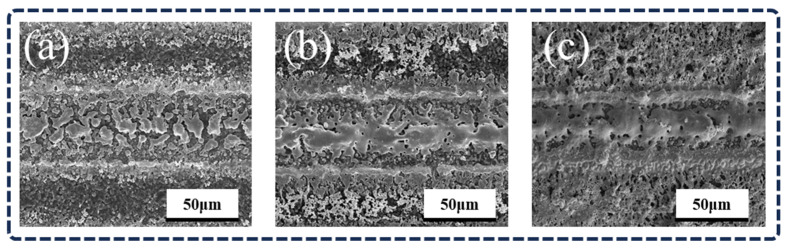
SEM images of (**a**) untreated sample; (**b**) after plasma treatment; and (**c**) after the addition of PVP-30.

To investigate the effects of substrate surface condition on the morphology of the deposited structures, plasma pretreatment was performed on the ceramic substrates, and the corresponding results are presented in Figure 13 and Figure 14b. After plasma treatment, the contact angle of the ceramic surface decreased from 48.25° to 19.05°. Compared with the untreated ceramic substrate shown in Figure 14a, the plasma-treated samples exhibited deposited structures with significantly improved continuity within the scanning region. The particle boundaries became less distinct and more interconnected, while the number of pores decreased significantly, resulting in a denser structure with enhanced connectivity.

This phenomenon can be attributed to the plasma treatment process, during which surface contaminants are decomposed into small molecules such as CO_2_ and H_2_O and subsequently removed by the vacuum system [27]. As a result, a clean ceramic surface is exposed, while nano- to micro-scale pits and grooves are generated, thereby increasing the surface area and roughness. In addition, polar functional groups are introduced onto the ceramic surface, leading to a significant enhancement in surface energy [28]. Therefore, plasma pretreatment enables effective surface cleaning, surface roughening, chemical activation, and functional modification of ceramic substrates, thereby providing more favorable interfacial conditions for subsequent deposition.

In addition, the deposition system was further optimized by introducing PVP-30 into the precursor solution, and the corresponding results are presented in Figure 14c. After the addition of PVP-30, the deposited bands gradually evolved into a more continuous and smoother morphology, and a relatively integrated strip-like structure was formed in the central region of the deposition area. Surface fluctuations became less pronounced, the edge contours became clearer, and the lateral diffusion effect was effectively suppressed. Consequently, the overall uniformity and compactness of the deposited structures were significantly improved, although a few localized microporous defects still remained.

These findings suggest that the role of PVP-30 is not merely associated with changes in particle size, but is primarily related to improving the film-forming capability and stability of the precursor liquid film. PVP-30 regulates the spreading behavior of the precursor solution on the ceramic surface and suppresses the random migration and agglomeration of silver particles during the reduction and deposition process. Meanwhile, it facilitates interparticle interconnection and coalescence, thereby enhancing the spatial confinement effect during laser-induced reduction. As a result, silver conductive structures with clearer boundaries, improved continuity, and higher compactness can be obtained.

### 3.3. Assessment of Adhesion Properties and Conductive Stability of Deposited Structures

The adhesion performance of the laser-deposited silver structures was evaluated using a tape-peeling test. Owing to the narrow linewidth and limited loading area of individual line-shaped silver structures, it was difficult to directly perform standard cross-hatch adhesion testing. Furthermore, local morphological inhomogeneity may cause the peeling results to be strongly influenced by random factors. Therefore, using a single silver structure directly as the testing sample cannot accurately reflect the actual interfacial bonding condition between the deposited layer and the substrate.

**Figure 15:**
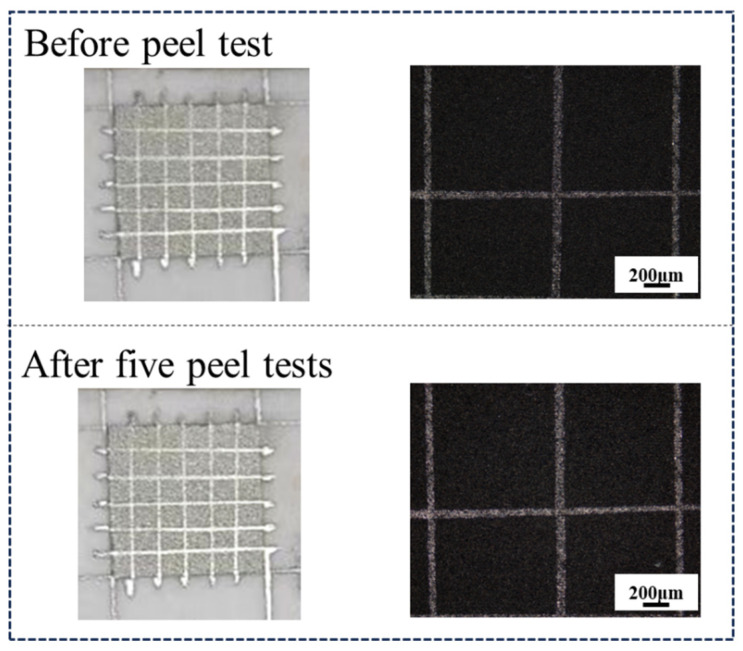
Deposited structures before and after five tape-peeling tests.

To enhance the reliability and comparability of the adhesion test results, the laser scanning spacing was reduced to 0.02 mm. Since the laser spot diameter of the employed laser system was approximately 0.023 mm, adjacent deposited lines partially overlapped, thereby forming a relatively uniform and continuous silver film region with dimensions of 6 mm × 6 mm. The fabricated silver film sample was subsequently used for adhesion performance evaluation. The corresponding test results are presented in Figure 15.

As illustrated in Figure 15, no noticeable peeling or detachment of the deposited structures was observed after the tape-peeling test, and no apparent loss of the silver film was detected in the localized regions. These results demonstrate that the metallic layer fabricated by the proposed method exhibits strong interfacial adhesion to the alumina ceramic substrate.

**Figure 16:**
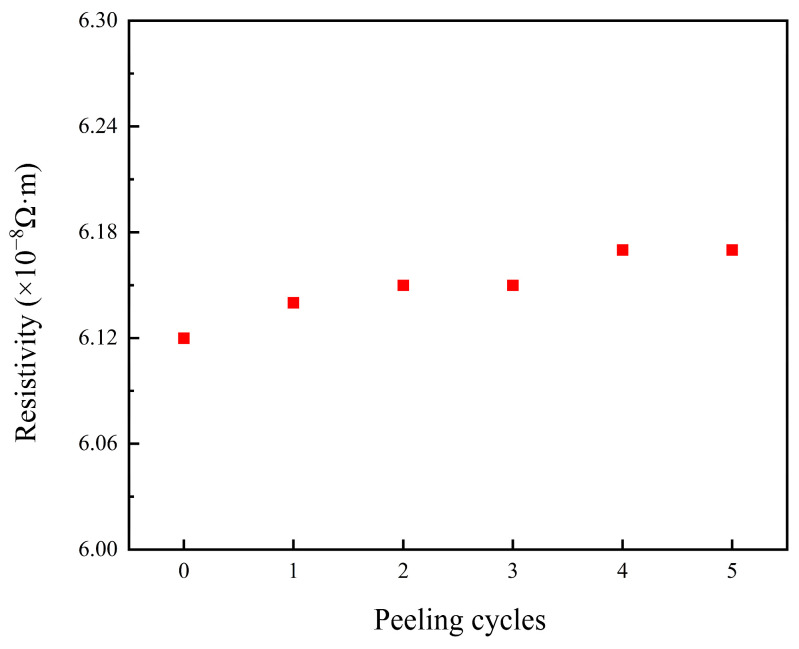
Resistivity of the deposited structures before and after five tape-peeling tests.

The deposited structures after the tape-peeling tests were further subjected to resistivity measurements, and the corresponding results are presented in Figure 16. After five peeling cycles, only a slight change in resistivity was observed, while the conductivity retention rate of the samples remained above 98%. The overall resistivity reached 6.14 × 10^−8^ Ω·m, which is of the same order of magnitude as the theoretical resistivity of silver (1.59 × 10^−8^ Ω·m). These findings indicate that the silver deposited structures prepared by this method exhibit both excellent interfacial adhesion and outstanding electrical conductivity.

## 4. Conclusions

(1) Silver conductive structures were fabricated in situ on 96% alumina ceramic substrates by selectively scanning a silver nitrate precursor liquid film using a 355 nm Nd:YAG nanosecond laser.
(2) The deposited structures were strongly influenced by the laser processing parameters and Ag^+^ concentration. Insufficient laser energy density resulted in incomplete Ag^+^ reduction and discontinuous conductive networks, whereas excessive energy input caused central hollow formation and Ag edge accumulation, thereby reducing structural uniformity and stability. A laser energy density of 12.03 J/cm^2^ provided a suitable balance among structural integrity, compactness, and electrical conductivity. Increasing the scan number promoted particle interconnection and conductive network formation, while approximately 1000 scanning cycles provided a relatively better balance among structural quality, dimensional precision, and electrical performance. As the AgNO_3_ concentration increased, the deposited structures evolved from discrete particles into continuous layers, with 5 mol/L showing favorable deposition performance under the investigated conditions.
(3) Interface engineering improved the continuity, compactness, and stability of the deposited structures. Plasma pretreatment reduced the contact angle of the ceramic surface from 48.25° to 19.05°, thereby enhancing surface wettability. The addition of PVP-30 improved the film-forming behavior and spatial confinement of the precursor liquid film, promoting particle interconnection and coalescence. The continuous silver film retained more than 98% of its conductivity after five tape-peeling tests, with a resistivity of 6.14 × 10^−8^ Ω·m. These results demonstrate that the proposed laser-induced deposition method enables the controllable fabrication of conductive silver structures on ceramic surfaces with good adhesion and electrical conductivity.

## Figures and Tables

**Table 1:** Chemical composition of the 96% alumina ceramic substrate.

Component	Al_2_O_3_	SiO_2_	MgO	P_2_O_3_	CaO
Content (wt.%)	95.8	1.8	1.4	0.8	0.2

**Table 2:** Laser Specifications.

Laser Parameters	Parameter Range
Wavelength (nm)	355
Pulse Width (ns)	15–60
Repetition Rate (kHz)	0.001–300
Average Laser Power (W)	≤2
Spot Diameter (μm)	23
Scanning Speed (mm/s)	1–2000
Scanning Area (mm)	100 × 100

## Data Availability

The datasets used or analyzed during the current study are available from the corresponding author upon reasonable request.

## References

[1] Fallah Nia E., Kouki A. (2024). Ceramics for microelectromechanical systems applications: A review. Micromachines.

[2] Feng J., Wang J., Liu H., Sun Y., Fu X., Ji S., Liao Y., Tian Y. (2024). A review of an investigation of the ultrafast laser processing of brittle and hard materials. Materials.

[3] Ramlow H., Ghasemi-Tabasi H., Burn A., Bayer M.H., Blugan G. (2025). Joining alumina to metals: Technologies, challenges, and future prospects for high-performance structures. J. Eur. Ceram. Soc..

[4] Tsai J.T., Lin S.T. (2013). Silver powder effectiveness and mechanism of silver paste on silicon solar cells. J. Alloys Compd..

[5] Lai Y.S., Lai S.S., Li Y.J., Lin H.J., Chiang T.H. (2021). Investigation of SiO_2_-B_2_O_3_-ZnO-Bi_2_O_3_ glass frits on the interface reaction of silver front contacts. J. Alloys Compd..

[6] Fang J., Fu R., Gu X., Zhang X., Li G. (2021). Characterization of glass insulating thick films with Ag conductors for multilayer packages. Materials.

[7] Baptista A., Silva F., Porteiro J., Míguez J., Pinto G. (2018). Sputtering Physical Vapour Deposition (PVD) Coatings: A Critical Review on Process Improvement and Market Trend Demands. Coatings.

[8] Borowski P., Myśliwiec J. (2025). Recent advances in magnetron sputtering: From fundamentals to industrial applications. Coatings.

[9] Kaziev A.V., Kolodko D.V., Lisenkov V.Y., Tumarkin A.V., Kharkov M.M., Samotaev N.N., Oblov K.Y. (2023). Cu Metallization of Al_2_O_3_ Ceramic by Coating Deposition from Cooled-and Hot-Target Magnetrons. Coatings.

[10] Han S.H., Kim B.Y., Lee J.K., Seo I., Kang H.W., Yoo M.J. (2022). Selective metallization of piezoelectric ceramics by laser-induced surface modification combined with electroless copper plating. Ceram. Int..

[11] Flacker A., Gomes G.C., Silva M.O., Teixeira R.C. (2022). Morphology behavior of copper films deposited after wet surface treatment on polished alumina. J. Braz. Chem. Soc..

[12] He H., Fu R., Wang D., Song X., Jing M. (2007). A new method for preparation of direct bonding copper substrate on Al_2_O_3_. Mater. Lett..

[13] Choi S.G., Kim S., Lee J., Kim K.S., Hyun S. (2025). Optimization of Direct Bonding Process for Lotus-Type Porous Copper to Alumina Substrates. J. Manuf. Mater. Process..

[14] Lee S.K., Tuan W.H., Wu Y.Y., Shih S.J. (2013). Microstructure–thermal properties of Cu/Al_2_O_3_ bilayer prepared by direct bonding. J. Eur. Ceram. Soc..

[15] Li X., Li H., Chen Y., Zeng X. (2004). Silver conductor fabrication by laser direct writing on Al_2_O_3_ substrate. Appl. Phys. A.

[16] Hui Y., Na Z.L., Sun B.M., Sun X.D., Chen J.L. (2023). A Novel laser scanning method for metallization of alumina substrate by copper. Rare Met..

[17] Cheng J., Yang Z., Jiang S., Li F., Liu D. (2023). Hybrid laser processes for thick silver coating fabrication on AlN substrate. Ceram. Int..

[18] Nedyalkov N., Stankova N., Padikova F., Valkov S., Atanasova G., Dilova T., Aleksandrov L. (2025). Resistive heater element based on a conductive line in AlN ceramic fabricated by laser processing. Materials.

[19] Setoura K., Ito S., Yamada M., Yamauchi H., Miyasaka H. (2017). Fabrication of silver nanoparticles from silver salt aqueous solution at water-glass interface by visible CW laser irradiation without reducing reagents. J. Photochem. Photobiol. A Chem..

[20] Wu J.T., Hsu S.L.C., Tsai M.H., Hwang W.S. (2010). Direct inkjet printing of silver nitrate/poly (N-vinyl-2-pyrrolidone) inks to fabricate silver conductive lines. J. Phys. Chem. C.

[21] Huang H.H., Ni X.P., Loy G.L., Chew C.H., Tan K.L., Loh F.C., Deng J.F., Xu G.Q. (1996). Photochemical formation of silver nanoparticles in poly (N-vinylpyrrolidone). Langmuir.

[22] (2023). Standard Test Methods for Rating Adhesion by Tape Test.

[23] Hu H., Larson R.G. (2006). Marangoni effect reverses coffee-ring depositions. J. Phys. Chem. B.

[24] Bremer S.J.L., Luckabauer M., Roemer G.W.R.B.E. (2023). Laser intensity profile as a means to steer microstructure of deposited tracks in directed energy deposition. Mater. Des..

[25] Pyeon J., Song K.M., Jung Y.S., Kim H. (2022). Self-Induced Solutal Marangoni Flows Realize Coffee-Ring-Less Quantum Dot Microarrays with Extensive Geometric Tunability and Scalability. Adv. Sci..

[26] Liu Y., Zhang X., Poyraz S., Zhang C., Xin J.H. (2018). One-Step Synthesis of Multifunctional Zinc-Iron-Oxide Hybrid Carbon Nanowires by Chemical Fusion for Supercapacitors and Interfacial Water Marbles. ChemNanoMat.

[27] Wang Z., Li Y., Zhang P., Wang F., Sun L., Bai Q., Zhu M., Wang B. (2025). Low-pressure plasma cleaning of organic contamination from chemically coated surfaces: A study combining experimental and molecular dynamics studies. R. Soc. Chem. Adv..

[28] Dufour T. (2023). From basics to frontiers: A comprehensive review of plasma-modified and plasma-synthesized polymer films. Polymers.

